# Supplementary material for: Structural insights for selective disruption of Beclin 1 binding to Bcl-2
Source: Commun Biol. 2023 Oct 24;6:1080. doi: 10.1038/s42003-023-05467-w (PMC10598227; doi:10.1038/s42003-023-05467-w)
Supplement: Supplementary file 2 — Supplementary Figures [file 42003_2023_5467_MOESM2_ESM.pdf]

Supplementary material for:

Structural insights for selective disruption of Beclin 1 binding to Bcl-2[&]

Yun-Zu Pan[1,2,3], Qiren Liang[2], Diana R. Tomchick[1], Jef K. De Brabander[2] and Josep Rizo[1,2,3*]

[1]Department of Biophysics, University of Texas Southwestern Medical Center, Dallas, Texas 75390, USA

[2]Department of Biochemistry, University of Texas Southwestern Medical Center, Dallas, Texas 75390, USA

[3]Department of Pharmacology, University of Texas Southwestern Medical Center, Dallas, Texas 75390, USA

[*]For correspondence: Jose.Rizo-Rey@UTSouthwestern.edu

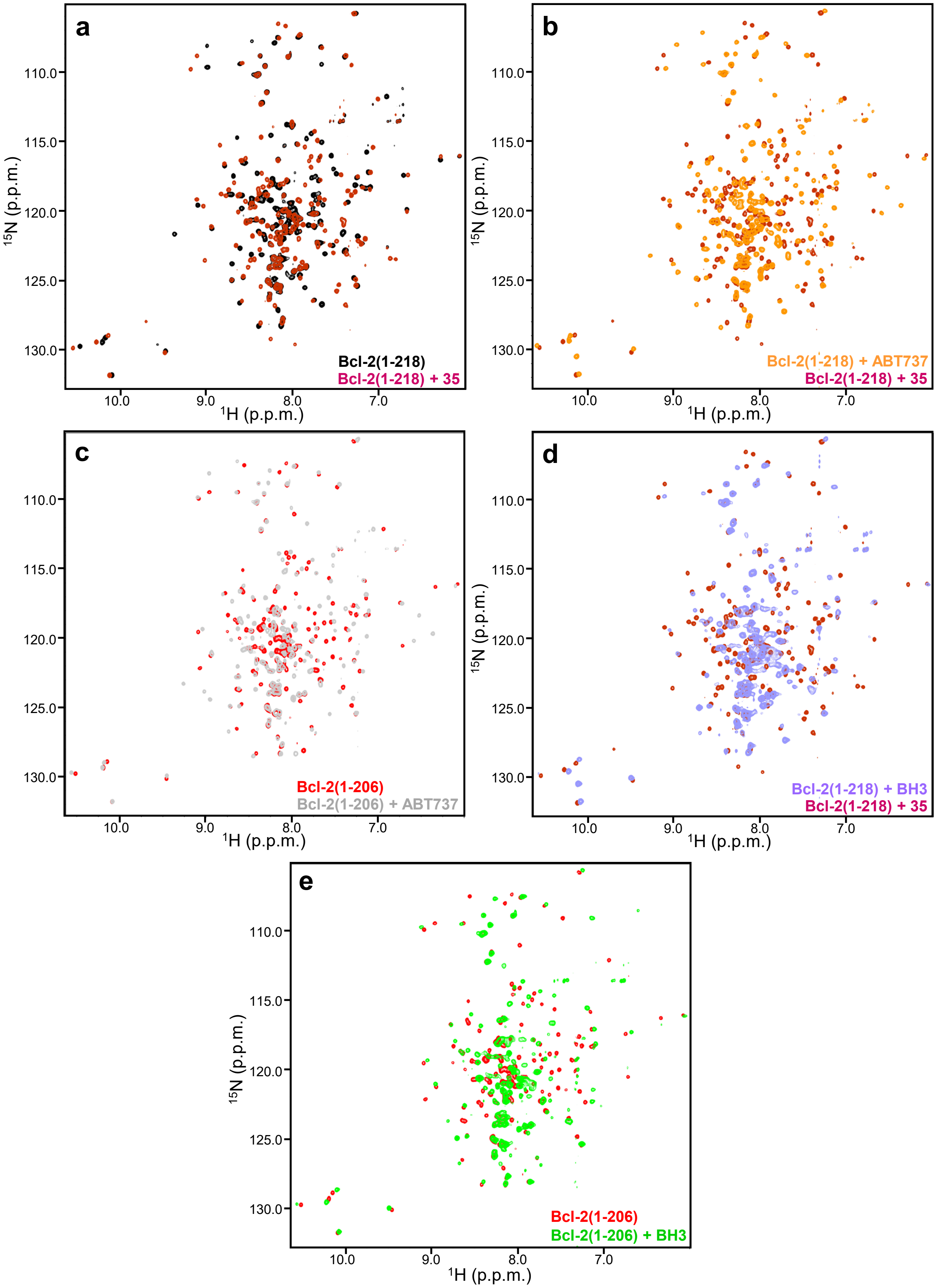

Figure S1
Pan et al.

Figure S1. Additional ^{1}H-^{15}N TROSY-HSQC spectra of Bcl-2(1-206) and Bcl-2(1-218). The contour plots show superpositions of ^{1}H-^{15}N TROSY-HSQC spectra of: (**a**) Bcl-2(1-218) in the absence and presence of **35**; (**b**) Bcl-2(1-218) in the presence of **35** or ABT-737; (**c**) Bcl-2(1-206) in the absence and presence of ABT-737; (**d**) Bcl-2(1-218) in the presence of **35** or Beclin 1 BH3 domain; and (**e**) Bcl-2(1-206) alone and in the presence of Beclin 1 BH3 domain. The contours plots are color coded as indicated by the labels at the bottom right corner.

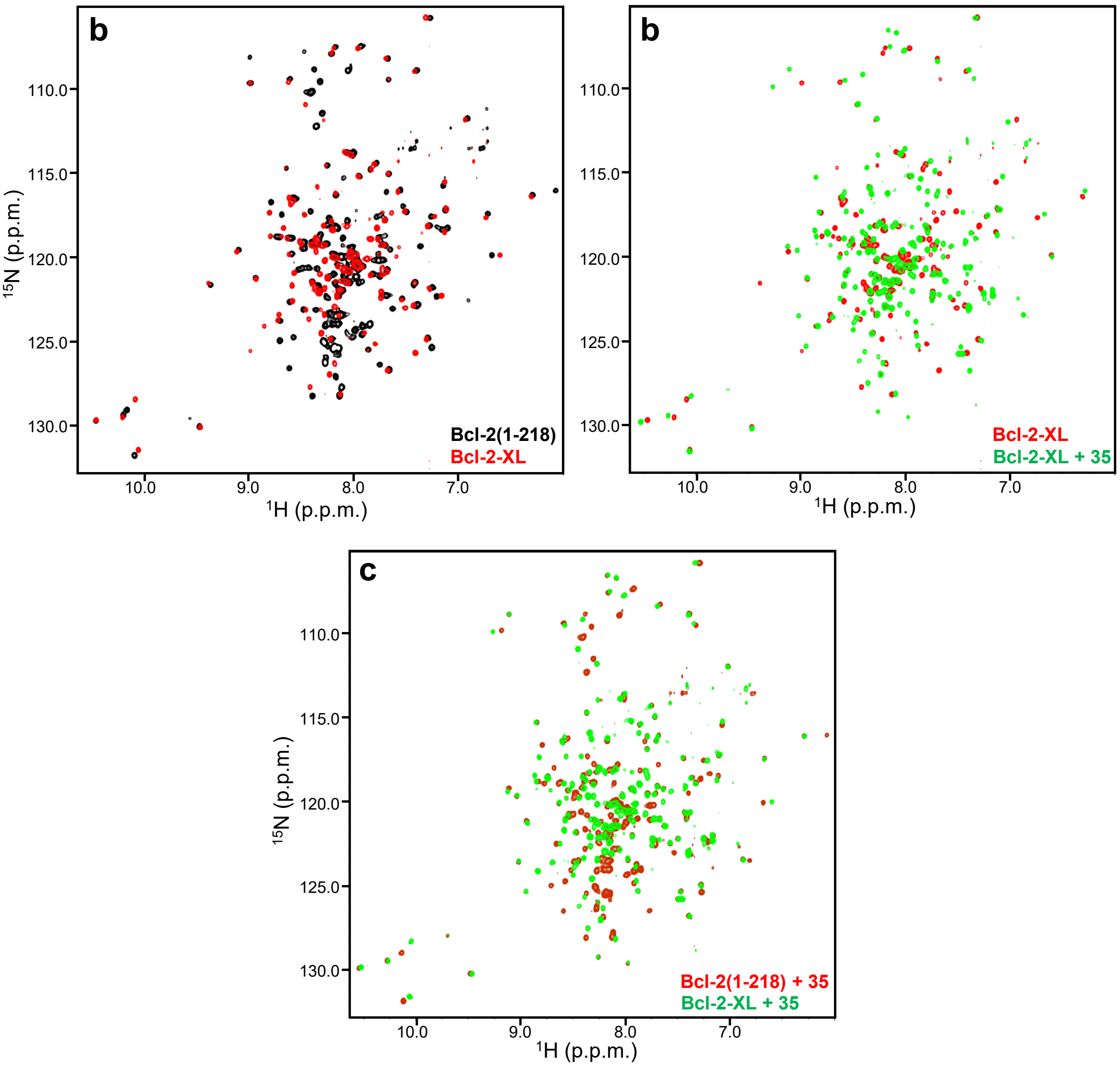

Figure S2
Pan et al.

Figure S2. Comparison of ^{1}H-^{15}N TROSY-HSQC spectra of Bcl-2(1-218) and Bcl-2-xL. The contour plots show superpositions of ^{1}H-^{15}N TROSY-HSQC spectra of: (**a**) isolated Bcl-2(1-218) and Bcl-2-xL; (**b**) Bcl-2-xL in the absence and presence of **35**; and (**c**) Bcl-2(1-218) and Bcl-2-xL in the presence of **35**. The contours plots are color coded as indicated by the labels at the bottom right corner.

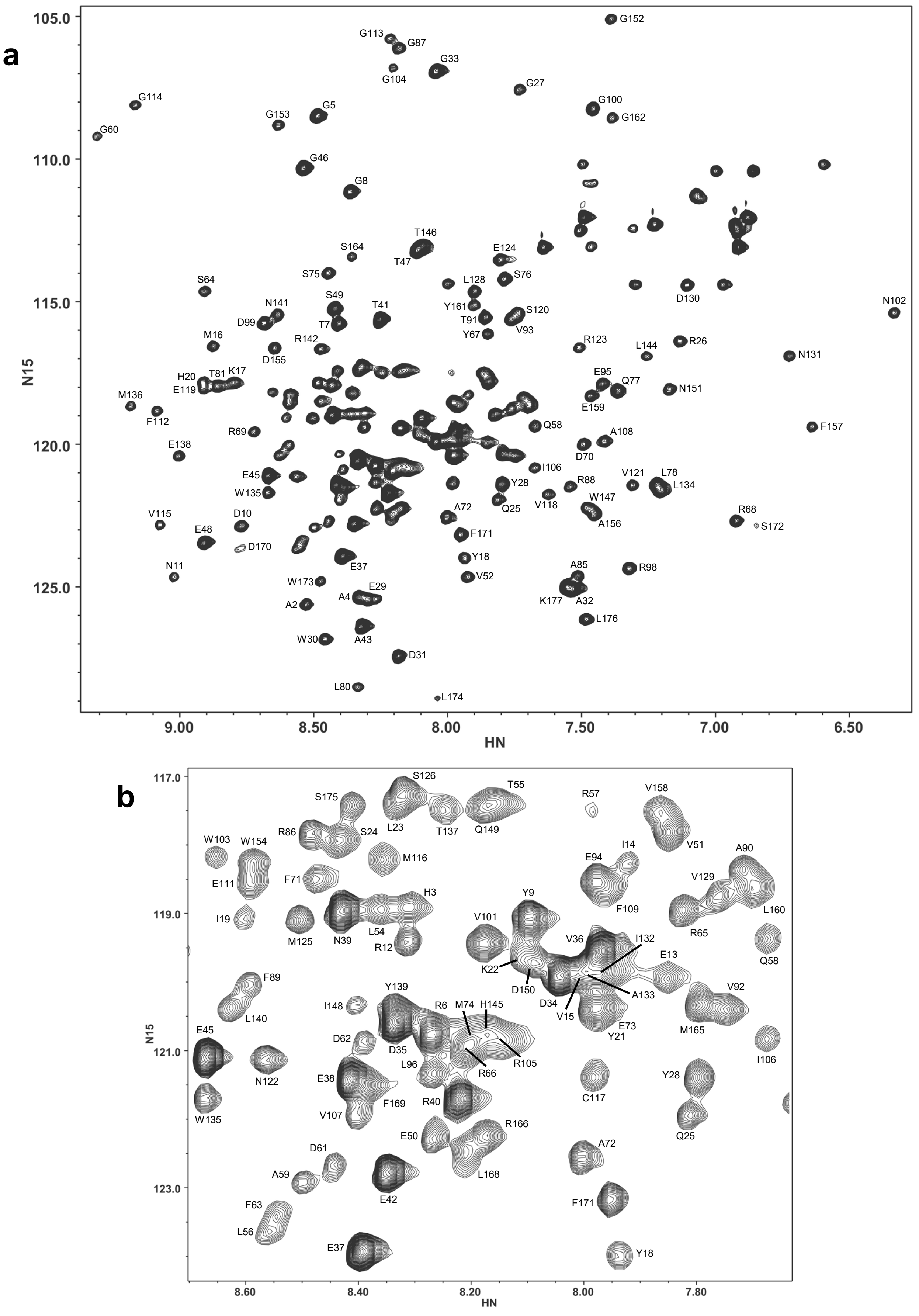

Figure S3
Pan et al.

Figure S3. Assignment of the ^{1}H-^{15}N HSQC spectrum of the Bcl-2-xL/**35** complex. (**a,b**) Expansions of an ^{1}H-^{15}N HSQC spectrum of the Bcl-2-xL/**35** complex indicating the assignments of the backbone crosspeaks.

Protein	Sequence	Residues
Beclin 1	GTMENLSRRLKVTGDLFDIMSGQTDVDHP	107-135
Bax	ASTKKLSECLKRIGDELDSNMELQRMIAA	54-82
Bak	STMGQVGRQLAIIGDDINRRYDSEFQTML	10-38
Bok	GRLAEVCAVLLRLGDELEMIRPSVYRNVA	61-89
Arf-BP1	VMTQEVGQLLQDMGDDVYQQYRSLTRQSS	1971-1999
Bid	DIIRNIARHLAQVGDSMDRSIPPGLVNGL	81-109
Bik	EGSDALALRLACIGDEMDVSLRAPRLAQL	52-80
Bim	RPEIWIAQELRRIGDEFNAYYARRVFLNN	143-171

Figure S4
Pan et al.

Figure S4. Sequence alignment of the BH3 domain of Beclin 1 and several pro-apoptotic proteins. All sequences are from *Homo Sapiens*. The residue numbers corresponding to each BH3 domain are indicated on the right. Note that there is only limited homology among the BH3 sequences and only a few residues are generally conserved (highlighted in yellow).

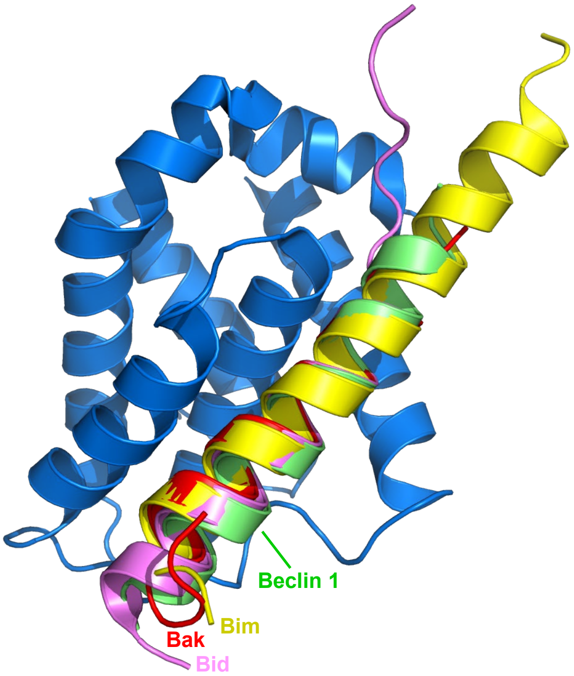

Figure S5
Pan et al.

Figure S5. Superposition of structures of complexes between Bcl-xL and various BH3 domains. A ribbon diagram of Bcl-xL (blue ribbon) bound to the Beclin 1 BH3 domain (lime ribbon) is superimposed with the structure of Bcl-xL bound to the BH3 domains from Bim (yellow), Bak (red) or Bid (violet) (PDB accession numbers 2P1, 1PQ1, 5FMJ and 2P1L, respectively). Bcl-xL is shown for only the Beclin 1 complex for simplicity.
